# Assessment of Family Planning Service Availability and Readiness in 10 African Countries

**DOI:** 10.9745/GHSP-D-18-00041

**Published:** 2018-10-03

**Authors:** Moazzam Ali, Madeline Farron, Thandassery Ramachandran Dilip, Rachel Folz

**Affiliations:** a Department of Reproductive Health and Research, World Health Organization, Geneva, Switzerland.; b University of Michigan School of Public Health, Ann Arbor, MI, USA.; c Department of Maternal, Newborn, Child, and Adolescent Health, World Health Organization, Geneva, Switzerland.

## Abstract

**Background:**

Access to family planning services and appropriate contraceptive methods is crucial for ensuring good health outcomes for women and adolescent girls. The World Health Organization worked with the U.S. Agency for International Development to develop the Service Availability and Readiness Assessment (SARA) survey to measure health facility capacity to provide end users with appropriate, high-quality health care. In this study, we looked at the service availability and readiness of health facilities to provide contraception in 10 African countries: Benin, Burkina Faso, the Democratic Republic of the Congo, Djibouti, Mauritania, Niger, Sierra Leone, Tanzania, Togo, and Uganda.

**Methods:**

This study compared SARA survey data on family planning services from each of the 10 countries. We conducted a descriptive analysis of variations in facility readiness and the availability of services, contraceptive methods, trained staff, family planning guidelines, and basic health care equipment.

**Results:**

Overall, many of the countries surveyed had a relatively high availability of at least 1 contraceptive method. Rural facilities tended to have more availability of contraception than urban facilities, and government facilities tended to have higher availability of family planning than other providers. The countries differed in their particular dominant contraceptive method, and stock-outs of contraceptive methods were observed. Countries had overall low levels of all 6 tracer items (availability of family planning guidelines, staff trained in family planning, blood pressure apparatuses, combined oral contraceptive, injectable contraceptives, and male condoms on the day of the assessment), indicating low health system readiness. There were discrepancies between reported and observed availability of blood pressure apparatuses and family planning guides and having at least 1 staff member trained to use these tools. In all countries, unmarried adolescents appeared to have less access to family planning than the general population.

**Conclusion:**

Stock-outs and logistics management problems were common among the countries surveyed. Critical gaps between reported and actual availability of products and services often makes it difficult for end users to access appropriate family planning methods. To address many of the issues, additional health worker training is needed and more effort to target and support adolescents should be undertaken. To achieve universal health coverage targets for family planning, gaps in the availability and readiness of health systems to provide contraceptive products and services must be reduced.

## Abstract (precis)

In the 10 countries surveyed, the availability of oral contraceptives, injectables, and condoms varied greatly, and the availability of basic items indicating service readiness, such as guidelines, trained staff, equipment, and certain commodities, was low.

Résumé en français à la fin de l'article.

## INTRODUCTION

In working to achieve the Sustainable Development Goals,^1^ it is important to be able to objectively monitor and evaluate the progress countries make as they implement new strategies. When working specifically toward health outcome-related goals, it is essential to measure the quality of health systems; however, this can be difficult. For this reason, the World Health Organization (WHO) has been working in coordination with the U.S. Agency for International Development (USAID) and other partners to develop a tool, called the Service Availability and Readiness Assessment (SARA) tool, that breaks down health systems into measurable, trackable components that can provide data about the progress of health systems strengthening efforts.^2^ The tool builds on previous and current approaches designed to assess health facility service delivery including the Service Availability Mapping (SAM) tool and the Service Provision Assessment (SPA) tool developed by ICF International under the USAID-funded Demographic and Health Surveys (DHS) Program.^3^ The SARA tool measures access to health systems. Access has 2 components: “availability,” which refers to and focuses on the physical presence or reach of the facilities and “readiness,” which examines the potential of health facilities to provide basic health care interventions relating to family planning, child health services, basic and comprehensive obstetric care, HIV/AIDS, tuberculosis, malaria, and noncommunicable diseases.^2^ The tool also assesses the availability of physical infrastructure and trained manpower within the a health system.^2^ While these components contribute to the quality of a health facility, they, of course, do not guarantee the delivery of quality services. However, the information gained from this tool can be used to fill critical data gaps about country service delivery mechanisms and health systems strengthening and to identify places where countries should invest resources to meet their health goals.

The SARA tool measures service readiness, i.e., physical access to and capacity of health facilities to provide health services.

The SARA tool is a questionnaire that was designed to measure country-specific health-related goals, including family planning. Before the tool is implemented, a country must first compile a master facility list to direct the use of the surveys. The survey can then be used to either randomly sample health facilities within a country or assess all facilities within selected local districts.^4^ Data collection is performed by specially trained survey teams, or by national ministries of health or national institutes. To complete the survey, the data collection team spends an average of 2 to 4 hours at a facility. The visit involves interviewing key informants and verifying the availability of supplies, equipment, medicines, and other commodities at the time of the visit.^4^ The survey information is then compiled and analyzed using standard SARA core indicators and any additional country-specific indicators that were specified in the evaluation plan. The results are then disseminated to national stakeholders and researchers.^2^

SARA is not intended to provide comprehensive data on all aspects of functioning of health services, rather, the tool focuses on key “tracer” items that are indicative of the essential health system underpinnings and crucial to programs that are scaling up or ready to do so. Family planning data from the SPA tool is more comprehensive—as it not only covers service availability and readiness but also examines client–provider interaction components of consultation, counseling and discussions, client knowledge levels, and feedback from family planning clients on service accessibility problems. In total, 10 sub-Saharan African countries have implemented the SARA tool since 2010, whereas only 3 countries have used the SPA tool during the same time frame.

The SARA tool has been successfully used to generate a detailed assessment of the status of full health systems in such countries as Uganda^5^; monitor and evaluate progress toward universal health coverage in South Africa^6^; and assess availability and readiness of health facilities to provide general and specific services, such as chronic disease management in Uganda,^7^ maternal and child health in Madagascar,^8^ or family planning and child immunization in Burkina Faso, Cambodia, Haiti, Sierra Leone, and Tanzania.^4^ The tool has also been valuable for making multi-country assessments and comparisons of reproductive, maternal, newborn, and child health to determine successful efforts toward meeting the Millennium Development Goals;^9^ assessing surgical availability and readiness in Benin, Burkina Faso, the Democratic Republic of the Congo (the DRC), Mauritania, Sierra Leone, Togo, and Uganda^10^; and achieving other research objectives.

The data provided by the SARA tool can be used to generate detailed health system assessments and monitor and evaluate progress toward universal health coverage.

## METHODS

In this study, we used data generated from SARA surveys conducted between 2010 and 2016 to assess and compare the availability and readiness of health facilities to provide patients with family planning services in 10 African countries, with the aim of generating further evidence for the planning and management of health systems.^1^ The 10 countries had volunteered to implement the SARA tool, with technical assistance from WHO and partners, in health facilities within their respective countries: Benin (2013, N=788 facilities), Burkina Faso (2014, N=766 facilities), Djibouti (2015, N=82 facilities), the DRC (2014, N=1,555 facilities), Mauritania (2016, N=288 facilities), Niger (2015, N=372 facilities), Sierra Leone (2013, N=455 facilities), Tanzania (2012, N=1,297 facilities), Togo (2012, N=100 facilities), and Uganda (2013, N=209 facilities).

Countries tend to do SARA every 2 years.^10^ The sampling frame for assessment of service readiness is the master facility list.^4^ This master list comprises all health care facilities, including public and private facilities as well as health centers and dispensaries, and includes information on such things as beds, staffing, and services available.^4^ If a master facility list does not exist or is incomplete, a preliminary list should be created.^4^

Countries implementing SARA used 1 of 2 different sampling methods: (1) a nationally representative simple/systematic random sample of health facilities—with stratification by type of health facility and managing authority^2^—to obtain national estimates or (2) a census of all facilities in selected districts, which can be used for subnational estimates if desired.^4^ Led by national ministries of health or national institutes, 2 surveyors on each survey team collected data using paper forms and the Census and Survey Processing System (CSPro) (U.S. Census Bureau and ICF International, Washington DC, USA) electronic data processing software package.^4^ On average, facility visits take approximately 2 to 4 hours. The visit involves interviewing key informants and verifying reported and observed availability of essential equipment, supplies, medicines, and other commodities.^4^ A database of the survey information is then generated through double entry of the same questionnaire and comparison of responses. The range and consistency checks performed before production of standard SARA tables using Microsoft Excel program and then disseminated to national stakeholders.^4^

The SARA indicators measure service availability, general service readiness, and service-specific readiness.^2^ Service availability encompasses the physical presence of the delivery of services, including health infrastructure, core health personnel, and service utilization, but does not include more complex data such as geographic barriers, travel time, and user behavior.^2^ Service availability is described with an index using the 3 areas of tracer indicators, with the indicators expressed as a percentage compared with target or benchmark, then taking the mean of the area scores.^2^ General service readiness is expressed with an index using the 5 general service readiness domains and then a score is associated with each domain based on the number of domain elements present, with an overall readiness score calculated with the mean of the 5 domains.^2^ Service-specific readiness looks at a health facility's ability to offer a specific service and capacity to provide that service to a user. This is measured with selected tracer items such as trained staff, guidelines, equipment, diagnostic capacity, medicine, and commodities.^2^

SARA indicators measure service availability, general service readiness, and service-specific readiness, using an index of specific tracer items.

SARA can be used to explore reported and actual percentages of facilities offering certain services in national health system. Here, the reported percentage refers to health facilities where SARA survey respondents reported that their respective health facility is providing oral contraceptives in service, which has implications for available stock. The actual percentage refers to health facilities where, on the day of the assessment, a SARA surveyor observed at least 1 valid stock of oral/injectable contraceptives in the service area or where supplies were routinely stored in the health facility. We defined the stock-out rate of a contraceptive method as the proportion of facilities providing family planning services where the SARA surveyor did not observe at least 1 valid stock of oral or injectable contraceptives in the service area or where supplies were routinely stored in the health facility on the day of the assessment.

The SARA survey-based indicators measure contraceptive method choice and stock-out of oral contraceptives, injectable contraceptives, and male condoms. Other family planning provider readiness indicators—in terms of number of trained staff, availability and use of guidelines on delivering family planning services, and availability of a blood pressure apparatus for use of family planning staff—are used for cross comparison. The data are disaggregated by type (government or nongovernment) and location (rural or urban) of the family planning provider, and analysis is performed. Under the family planning section, there is a set of questions inquiring whether the facility provides or prescribes any of the following modern methods of family planning for unmarried adolescents: combined estrogen-progesterone oral contraceptive pills (COCs), male condoms, emergency contraceptive pills, and intrauterine devices (IUDs). We compared information on proportion of facilities providing these methods for unmarried adolescents across the countries.

## RESULTS

### Facilities Providing Contraceptives by Type of Contraceptive Offered

Niger (96%), closely followed by Sierra Leone (94%), had the highest proportion of health facilities offering family planning services (Table 1), while the DRC (33%), Djibouti (57%), and Mauritania (67%) had the lowest proportion. Very few countries had high availability of more than 1 contraceptive type. In general, COCs, male condoms, and progestin-only injectable contraception were offered at higher rates (Table 1). Burkina Faso, Niger, and Sierra Leone had the most contraceptive options available at their facilities, suggesting that more options are available across facilities in those countries. When looking at national-level data, Djibouti and the DRC had the fewest options available at their facilities. Implants had relatively high availability in some countries (Benin, Burkina Faso, and Niger) and low availability elsewhere (Djibouti, the DRC, Mauritania, Sierra Leone, Tanzania, Togo, and Uganda).

**TABLE 1.** Percentage of Facilities Providing Contraceptives, by Type of Contraceptive Offered

Country	Year of SARA Survey	Provides FP Services (%)	COCs (%)	POPs (%)	CICs (%)	POIs (%)	Male Condoms (%)	Female Condoms (%)	IUDs (%)	Implants (%)	CycleBeads for SDM (%)	ECPs (%)	Male Sterilization (%)	Female Sterilization (%)	Total No. of Facilities
Benin	2015	83	72	46	68	53	64	18	64	71	44	21	1	2	788
Burkina Faso	2014	91	89	80	NA	89	87	83	49	81	80	78	3	4	766
Djibouti	2015	57	50	49	32	30	45	23	29	12	1	37	1	2	82
DRC	2014	33	23	14	12	19	28	18	9	11	16	7	1	3	1,555
Mauritania	2016	67	64	55	44	59	55	28	20	29	3	9	NA	NA	288
Niger	2015	96	95	91	51	93	85	61	48	86	9	15	1	2	372
Sierra Leone	2013	94	89	85	33	79	92	79	25	33	7	43	1	1	455
Tanzania	2012	83	68	63	37	54	68	9	18	23	26	43	6	8	1,297
Togo	2012	84	65	44	38	66	65	16	40	46	31	17	NA	4	100
Uganda	2013	92	85	59	5	89	84	14	28	30	10	68	13	15	209

Abbreviations: CICs, combined injectable contraceptives; COCs, combined oral contraceptives; DRC, Democratic Republic of the Congo; ECPs, emergency contraceptive pills; FP, family planning; IUD, intrauterine device; NA, not available; POIs, progestin-only injectables; POPs, progestin-only pills; SARA, Service Availability and Readiness Assessment; SDM, Standard Days Method.

In most countries, COCs, male condoms, and progestin-only injectables were offered at higher rates than other methods.

### Location and Type of Provider

**FIGURE 1:**
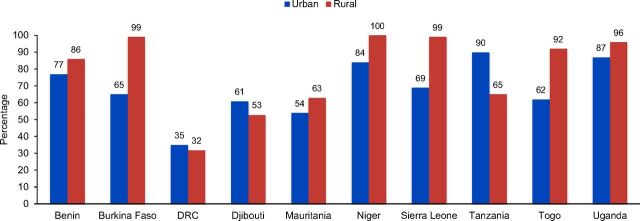
Percentage of Health Facilities Providing Family Planning, by Urban and Rural Location Abbreviation: DRC, Democratic Republic of the Congo.

**FIGURE 2:**
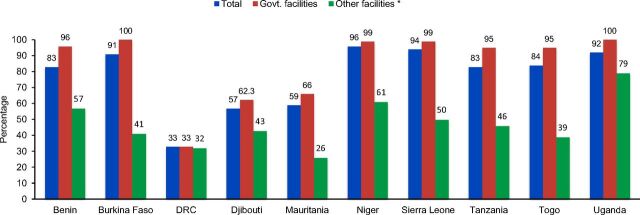
Percentage of Health Facilities Providing Family Planning Services, by Type of Facility Abbreviation: DRC, Democratic Republic of the Congo. * “Other facilities” includes all health care providers not managed by the government, including private sector, faith-based organizations, NGOs, and other similar organizations.

Breakdowns of country-level urban and rural family planning availability showed that in 8 of the 10 countries—Benin, Burkina Faso, Mauritania, Niger, Sierra Leone, Tanzania, Togo, and Uganda—rural facilities had a higher availability of family planning services than urban facilities (Figure 1). Likewise, a comparison of government facilities versus other facilities—those managed by private sector, faith-based organizations, NGOs, and any others—was consistent across countries, with all 10 countries having higher percentages of family planning availability in government facilities compared with other facilities (Figure 2).

In 8 of the countries, rural facilities had a higher availability of family planning services than urban facilities.

### Stock-Outs on the Day of the Assessment

**FIGURE 3:**
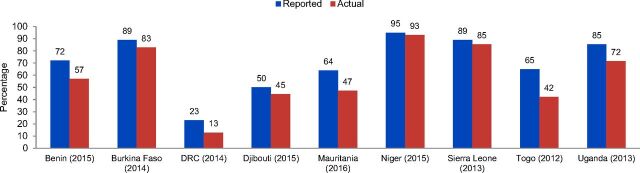
Percentage of Facilities Providing Oral Contraceptives, Reported Compared With Observed^a^ Abbreviation: DRC, Democratic Republic of the Congo. ^a^ Reported percentage refers to percentage of facilities where a SARA survey respondent reported that a health facility is providing oral contraceptive services. Actual percentage refers to the percentage of facilities where a SARA surveyor observed at least 1 valid stock of oral contraceptives in the service area or in a place where they are routinely stored in the health facility, on the day of the assessment

**FIGURE 4:**
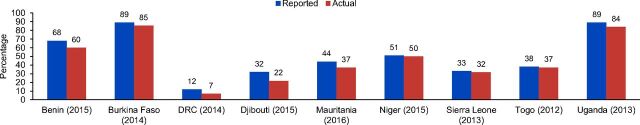
Percentage of Facilities Providing Injectable Contraceptives, Reported Compared With Observed^a^ Abbreviation: DRC, Democratic Republic of the Congo. ^a^ Reported percentage refers to percentage of facilities where a SARA survey respondent reported that a health facility is providing oral contraceptive services. Actual percentage refers to the percentage of facilities where a SARA surveyor observed at least 1 valid stock of oral contraceptives in the service area or in a place where they are routinely stored in the health facility, on the day of the assessment.

Among the 10 countries, stock levels varied depending on the type of method and the location or type of facility providing family planning methods (Figure 3, Figure 4, and Supplement Table for more details). Burkina Faso, Niger, and Sierra Leone seemed to have consistently high levels of stock available, with a good mix of 3 options available at similarly high rates. In contrast, Togo had a large disparity between injectables with high amounts in stock compared with oral contraceptives and male condoms, and the DRC had a disparity between higher levels of male condoms being in stock compared with oral contraceptives or injectables. Among the 10 countries, government-run facilities tended to have a higher percentage of contraceptive methods in stock—condoms being the highest percentage stocked—compared with other facilities. Both types of facilities seemed to have similar percentages of oral contraceptives, injectables, and male condoms in stock. Whether a facility was urban or rural did not seem to affect availability of stock; however, facility-type data showed “government facilities” generally had more stock for each type of contraceptive compared with “other facilities,” with some exceptions. Stock-out rates also tended to be higher in countries with lower proportions of facilities providing family planning services.

**FIGURE 5:**
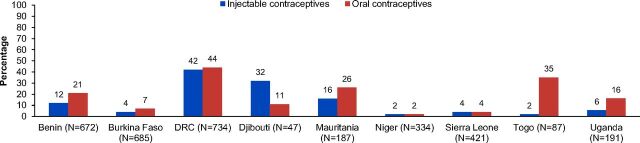
Percentage of Health Facilities With Stock-Outs of Injectable and Oral Contraceptives Abbreviation: DRC, Democratic Republic of the Congo. Percentage stock-out refers to the proportion of facilities providing family planning services, where a SARA surveyor did not observe at least 1 valid stock of injectable or oral contraceptives in the service area or in the place where they are routinely stored in the health facility, on the day of the assessment.

Another feature of SARA is the ability to explore reported and actual percentages of facilities offering certain services, which has implications for available stock. As mentioned earlier, actual availability refers to whether a family planning method was observed in the service area or in the place they are routinely stored on the date of SARA survey. Data for the reported versus actual percentage of facilities providing oral contraceptives in 9 of the countries—Benin, Burkina Faso, Djibouti, the DRC, Mauritania, Niger, Sierra Leone, Togo, and Uganda—show the proportion of health facilities experiencing stock-out of oral contraceptives ranged from 2% in Niger to 35% in Togo (Figure 5). The stock-out rate in health facilities for relatively less popular injectable contraceptives ranged from 2% in Niger and Togo to 42% in the DRC. The DRC, Mauritania, and Togo also had high stock-out rates for oral contraceptives among their facilities. Data from these same 9 countries show disparities between actual and reported percentages of facilities providing injectable contraceptives and oral contraceptives (Figure 5).

**FIGURE 6:**
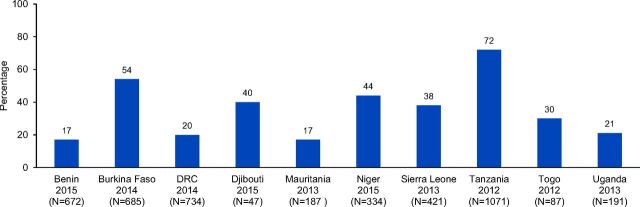
Percentage of Facilities Providing Family Planning Services With All Tracer Items^a^ Abbreviation: DRC, the Democratic Republic of the Congo. ^a^ Tracer items include availability of guidelines for family planning, staff trained in family planning services, blood pressure apparatus, combined oral contraceptive pills, injectable contraceptives, and male condoms on the day of facility assessment.

A key feature of SARA is its ability to pool data on tracer items. The 6 tracer items identified for family planning include the availability of and access to guidelines/job aids for family planning, staff trained in family planning services, blood pressure apparatuses, COCs, injectable contraceptives, and male condoms on the day of facility assessment. The percentage of facilities providing family planning services with all 6 tracer items ranged from 17% in Benin and Mauritania to 72% in Tanzania (Figure 6). The mean percentage of facilities providing family planning services with all 6 tracer items among the 10 countries was 35.3%.

The percentage of facilities providing family planning services with all 6 tracer items ranged from 17% in Benin and Mauritania to 72% in Tanzania.

### Guidelines, Equipment, and Training

Access to equipment and training shows not only the availability but also the readiness of a health service. At the health facilities surveyed, Mauritania and Uganda had the lowest percentage of family planning guidelines available, while Burkina Faso had the highest (Table 2). Facilities in all countries surveyed, except Sierra Leone and Uganda, had family planning guidelines (including checklists and/or job aids) available to inform family planning service provision at health facilities and had 1 or more staff members trained in family planning. Niger and Burkina Faso had relatively high levels of family planning guidelines available and staff members trained.

**TABLE 2.** Percentage of Facilities Providing Family Planning Services With Guidelines, Trained Staff, and Equipment

Country	Year	FP Guidelines (%)	At Least 1 Trained FP Staff (%)	BP Apparatus (%)	All 3 Items (%)
Benin	2015	64	47	96	35
Burkina Faso	2014	87	78	98	68
Djibouti	2015	74	66	87	55
DRC	2014	53	51	85	33
Mauritania	2016	44	54	91	NA
Niger	2015	82	78	83	52
Sierra Leone	2013	59	75	83	42
Tanzania	2012	57	45	89	NA
Togo	2012	67	42	88	36
Uganda	2013	44	49	86	28

Abbreviations: BP, blood pressure; DRC, Democratic Republic of the Congo; FP, family planning; NA, not available.

All 10 countries had a relatively high availability of blood pressure apparatuses in health facilities. Overall, the percentage availability of blood pressure apparatuses was highest in facilities, while the percentage of available family planning guidelines was much lower (Table 2). In 6 of 10 countries, 50% or more of facilities had more than 1 staff member trained in family planning, and in only 3 of 8 countries with data did 50% or more of facilities have all 3 family planning-related tracer items.

### Family Planning Services for Unmarried Adolescents

**FIGURE 7:**
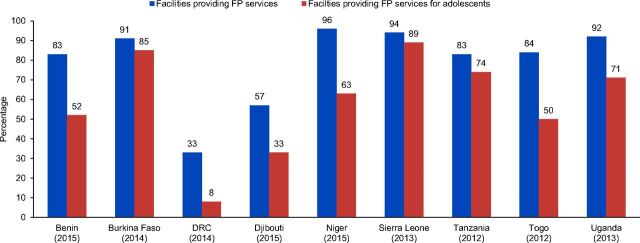
Percentage of Health Facilities Providing Family Planning Services to Unmarried Adolescents Abbreviations: DRC, Democratic Republic of the Congo; FP, family planning.

Adolescents are of particular interest when it comes to family planning services. The SARA tool includes separate questions about the provision of condoms and at least 1 other method of family planning to unmarried adolescents. Information provided in this section is used for comparison on availability of family planning services in general in the facility and specific availability of services for unmarried adolescents. For this metric, 9 countries were examined (data for Mauritania were not available). In 7 countries, the percentage of facilities providing family planning services was considerably higher than the percentage of facilities offering family planning services for unmarried adolescents, with Benin (31% difference), Niger (33% difference), and Togo (34% difference) having the biggest disparities (Figure 7). Burkina Faso and Sierra Leone had nearly the same level of services available to unmarried adolescents as the general public.

While the percentage of facilities providing family planning services to unmarried adolescents varied greatly, adolescents remained underserved.

## DISCUSSION

The SARA tool provided insightful information on the availability of family planning products and services as well as the readiness of the facilities surveyed to provide services in the 10 countries surveyed. While the results indicated overall high levels of family planning availability of at least 1 method in 7 countries (Djibouti, the DRC, and Mauritania did not have high levels of family planning availability), they also showed that contraceptive options were limited in the countries assessed, which could limit usage of family planning if appropriate methods are not available.

Despite results indicating overall high levels of family planning availability of 1 method in the countries assessed, contraceptive options were limited.

Results also showed that government health facilities typically had more family planning availability, highlighting potential gaps where public facilities do not exist. The difference between rural and urban family planning availability noted here could be due to a concentration of private facilities in urban areas, which is to be explored separately. The delivery of family planning services may not be a priority area for the profit-oriented private sector, which could have indirectly contributed to the observed differences between rural and urban service availability.

The typically higher stock levels in government facilities may be due to the government having better logistics management and/or supply chains than religious, private, or traditional health facilities. As a result, modern contraception may not be offered or considered as high of a priority in nongovernment facilities.

Stock-outs are a common problem. The 10 countries had varying stock levels and inconsistent method availability of the 3 methods surveyed: oral contraceptives, injectables, and male condoms. Some countries, particularly the DRC, had very high stock-out rates for certain commodities. In contrast, Burkina Faso, Niger, and Sierra Leone had the best stock mix of the countries surveyed. Poor levels of stock may be indicative of larger supply chain problems, such as poor logistics management information systems, incorrect ordering of stock, poor budget allocation and/or use, external supply chain issues, commercial market factors, or other transportation-related issues. Other factors, such as demand or intention, may have also influenced the different stock levels of these 3 methods in each of the surveyed countries. For example, a facility may stock male condoms for sexually transmitted infection prevention rather than family planning. Male condoms also do not offer women the same control or efficacy as other methods for family planning purposes. More worrisome are the countries such as the DRC, Mauritania, and Togo, which had lower levels of stock in general compared with the other countries surveyed. Stock-out rates were higher in countries with lower proportions of facilities—the DRC and Mauritania—providing family planning services, which may indicate that family planning is less of a priority for the government and/or public.

The differences between reported and actual percentages of facilities offering family planning services indicate that each country had a disparity in their oral and injectable contraceptive supplies. This may be due to either weak logistics management information systems and inadequate information about inventory and procurement or incorrect ordering of inventory by staff members who may have not received proper training for procurement or documenting inventory, or both. Ministries of health should prioritize improving logistics management information systems to have more accurate inventories to improve family planning and other health services.

Readiness is an area where many of the countries surveyed could use improvement. Overall low levels of tracer items—guidelines for family planning, staff trained in family planning services, blood pressure apparatus, COCs, injectable contraceptives, and male condoms on the day of facility assessment—indicated that the readiness of many health facilities in these countries needs to be improved. The percentage of facilities with guidelines for family planning and at least 1 staff member trained in family planning was low in the countries surveyed. In each country, except for Sierra Leone and Uganda, family planning guidelines were more likely to be in a health facility than staff members trained in family planning. This is a cause of concern because even if guidelines are available in a facility, they may not be used if staff members are not trained to use them. Even greater disparities were seen between the high availability of blood pressure apparatuses and the share of family planning service delivery units equipped with blood pressure apparatuses. We only have information on mere availability of the apparatus, and not on whether the providers are effectively using them. However, as noted in WHO's latest *Selected Practice Recommendations for Contraceptive Use*,^11^ while it is desirable to have blood pressure measurements taken, women should not be denied use of contraceptive methods simply because their blood pressure cannot be measured.

Overall low levels of tracer items indicated that the readiness of many health facilities in these countries should be improved.

According to SARA data, unmarried adolescents, in particular, are underserved. The data from Benin, Djibouti, the DRC, Niger, Togo, and Uganda showed especially low percentages of facilities with family planning services available for unmarried adolescents, which is a missed opportunity for country programs. By meeting the unmet need for modern contraception of women aged 15 to 19 years, countries could reduce unintended pregnancies by 6 million annually, thereby averting 2.1 million unplanned births, 3.2 million abortions, and 5,600 maternal deaths.^12^ Beyond that, reducing unintended pregnancies in adolescents can decrease the negative consequences of early childbearing—such as high-risk pregnancies that can cause complications and poor health outcomes for both mother and newborn child—and increase savings in maternal and child health care and improve young women's education and economic prospects.^11^

By meeting adolescents' unmet need for modern contraception, countries could avert 2.1 million unplanned births, 3.2 million abortions, and 5,600 maternal deaths.

At present, the core SARA tool focuses on measuring the availability of national guidelines, training of service providers, and availability of selected contraceptive methods, such as COCs, condoms, IUDs, and emergency contraception. The nature of information on adolescents in facility-level surveys is dependent on national policies and/or guidelines for providing family planning services for adolescents, which may vary from country to country. It is important to note that if a national policy does not support providing specific services to adolescents, then health facilities are not legally eligible to deliver them. In the absence of a national policy recommending provision of family planning method for adolescents, adolescent-specific service information is difficult to obtain. This is an inherent limitation in performing a cross-national comparison of adolescent access to various family planning methods using data from health facility surveys, including SARA.

### Limitations

While SARA data can produce many useful findings, it is important to acknowledge its limitations. The data only provide information about facility availability and readiness; they do not measure actual use of contraceptives. Moreover, assessing the supply side of family planning does consider potential low demand for family planning in general or for certain methods specifically, and how facility availability or readiness to provide family planning services may be affected in a place where demand may not exist. The information obtained on the availability of family planning services for unmarried adolescents was collected by interviewing 1 or more staff members at the facility. The interviews were not in-depth or robust and may have resulted in the actual availability being less than the stated results, possibly due to courtesy bias. It is crucial to review and revise data collection methods, as suggested above, to obtain good quality and credible information for strengthening services.

This analysis is limited, as it only looks at SARA data from 10 countries in sub-Saharan Africa and cannot necessarily be generalized to other countries or regions, and because long-acting reversible contraceptives (LARCs) and permanent methods were not included in the survey focus. The analysis was focused on short-acting reversible methods—oral contraceptives, injectables, and condoms—as the tracer for medicine and commodity availability for family planning services in SARA tool. Considering the increasing popularity of LARCs in sub-Saharan Africa, core SARA instruments may also consider adding them to the existing tracer items list.

The SARA tool should be revised to include LARCs and permanent methods to more accurately reflect the method mix available in sub-Saharan African countries.

## CONCLUSION

By improving the availability of an appropriate contraceptive method mix through supply-chain and logistics solutions and increasing facility readiness through training and improved health systems, more women could gain access to contraception thereby reducing an already identified unmet need.

Reducing gaps in availability and readiness of health systems to provide contraceptive products and services is needed to achieve universal health coverage targets for family planning. As discussed in this article, addressing time- and resource constraints-related limitations by expanding the scope of the core SARA tool can make it more programmatically useful for family planning planners and managers.

## Supplementary Material

18-00041-Ali-SupplementTable.pdf

## Notes

**En français**

**Évaluation de la disponibilité et de la capacité opérationnelle des services de planification familiale dans 10 pays africains**

Dans les 10 pays étudiés, la disponibilité des contraceptifs oraux, des contraceptifs injectables et des préservatifs est apparue très variable, tandis que la disponibilité d'indicateurs de base de la capacité opérationnelle (par exemple, lignes directrices, personnel qualifié, le matériel et produits spécifiques) était faible.

**RÉSUMÉ**

**Contexte général** : L'accès aux services de planification familiale et à des méthodes de contraception appropriées est indispensable pour obtenir des bons résultats sanitaires pour les femmes et les adolescentes. L'Organisation mondiale de la Santé (OMS) et l'Agence des États-Unis pour le développement international (USAID) ont mis au point conjointement l'outil de mesure de la disponibilité et de la capacité opérationnelle des services (SARA), qui sert à mesurer la capacité des établissements de santé à fournir aux utilisateurs finaux des soins appropriés et de qualité. Dans cette étude, nous nous sommes intéressés à la disponibilité des services et à la capacité opérationnelle des établissements de santé à fournir des services de contraception dans 10 pays africains : le Bénin, le Burkina Faso, Djibouti, la Mauritanie, le Niger, l'Ouganda, la République démocratique du Congo, la Sierra Leone, la Tanzanie et le Togo.

**Méthodes** : Cette étude a comparé les données des enquêtes SARA sur les services de planification familiale qui ont été menées dans chacun des 10 pays. Nous avons mené une analyse descriptive des variations observées pour la capacité opérationnelle des établissements et du point de vue de la disponibilité des services, des méthodes de contraception, du personnel qualifié, des lignes directrices sur la planification familiale, et du matériel de soins de base.

**Résultats** : Globalement, dans une grande partie des pays étudiés, au moins une méthode de contraception est très largement disponible. Les contraceptifs sont généralement plus disponibles dans les établissements ruraux que dans les établissements urbains, et les services de planification familiale plus accessibles dans les établissements publics que chez d'autres prestataires. La méthode de contraception principale diffère d'un pays à l'autre, et des ruptures de stock de moyens de contraception ont été observées. Globalement, les niveaux étaient bas pour 6 indicateurs (disponibilité de lignes directrices sur la planification familiale, personnel qualifié de planification familiale, appareils de mesure de la tension artérielle, contraceptifs oraux combinés, contraceptifs injectables, et préservatifs masculins le jour de l'évaluation), indiquant un faible niveau de préparation opérationnelle. Des écarts ont été notés entre la disponibilité signalée et observée des appareils de mesure de la tension artérielle, des guides de planification familiale et d'au moins un membre de l'équipe formé à l'usage de ces outils. Dans tous les pays, les adolescents non mariés ont moins accès à la planification familiale que la population générale.

**Conclusion** : Les ruptures de stock et les problèmes de gestion logistique sont courants dans les pays étudiés. Les écarts entre la disponibilité notifiée et effective des produits et des services complique souvent l'accès des utilisateurs finaux à des méthodes appropriées de planification familiale. Face à une grande partie de ces problèmes, des efforts supplémentaires de formation des agents de santé sont nécessaires et il faut mieux cibler et soutenir les adolescents. Pour atteindre les cibles de la couverture sanitaire universelle en matière de planification familiale, il faut réduire les lacunes observées en matière de disponibilité des produits et des services de contraception et du point de vue de la préparation opérationnelle des établissements.

## Notes

**Peer Reviewed**

**Funding:** The research was funded by the UNDP-UNFPA-UNICEF-WHO-World Bank Special Programme of Research, Development and Research Training in Human Reproduction, a cosponsored program executed by the World Health Organization.

**First Published Online:** September 13, 2018
