# Supplementary material for: Assessment of Family Planning Service Availability and Readiness in 10 African Countries
Source: Glob Health Sci Pract. 2018 Oct 3;6(3):473–83. doi: 10.9745/GHSP-D-18-00041 (PMC6172130; doi:10.9745/GHSP-D-18-00041)
Supplement: 18-00041-Ali-SupplementTable.pdf [file 18-00041-Ali-SupplementTable.pdf]

Ali M, Farron M, Dilip TR, Folz R. Assessment of family planning service availability and readiness in 10 African countries. *Glob Health Sci Pract*. 2018;6(3).
https://doi.org/10.9745/GHSP-D-18-00041

SUPPLEMENT TABLE. Percentage of Health Facilities With Stock[a] of Oral Contraceptives, Injectables, and Male Condoms on the Day of SARA Survey, by Type and Location of Facility

Country	Year	Percentage With Stock of Oral Contraceptives					Percentage With Stock of Injectables					Percentage With Stock of Male Condoms				
		All FP Facilities	Type of Facility		Location of Facility		All FP Facilities	Type of Facility		Location of Facility		All FP Facilities	Type of Facility		Location of Facility	
			Govt.	Others	Urban	Rural		Govt.	Others	Urban	Rural		Govt.	Others	Urban	Rural
Benin	2015	79	81	72	83	77	87	89	83	89	87	75	76	70	75	75
Burkina Faso	2014	93	96	57	76	97	96	98	64	83	99	94	98	64	83	99
Djibouti	2015	89	87	100	96	80	68	66	78	78	55	81	79	89	96	60
DRC	2014	56	54	60	63	54	58	57	61	76	54	80	81	79	86	78
Mauritania[b]	2013	68	71	33	62	73	57	59	33	56	58	61	64	35	61	62
Niger	2015	98	100	70	93	100	98	99	74	94	99	90	92	63	85	92
Sierra Leone	2013	96	97	90	95	96	96	97	85	92	97	98	99	88	93	99
Tanzania	2012	79	82	58	80	78	75	77	56	75	74	88	91	72	87	89
Togo	2012	65	66	56	78	61	98	100	78	89	100	66	68	47	84	61
Uganda	2013	84	88	75	82	85	94	99	84	92	96	84	84	84	88	81

Abbreviations: DRC, Democratic Republic of the Congo; FP, family planning; SARA, Service Availability and Readiness Assessment.

[a] "Stock" refers to the percentage of health facilities where a SARA surveyor observed at least 1 valid stock of contraceptives (oral pills, injectables, or condoms) in the service area or where they are routinely stored in the health facility on the day of the assessment.

[b] 2013 data is presented for Mauritania as their 2016 SARA summary report did not have this level of disaggregation.
